# Recent advances in *Entamoeba* biology: RNA interference, drug discovery, and gut microbiome

**DOI:** 10.12688/f1000research.9241.1

**Published:** 2016-10-26

**Authors:** Pedro Morgado, Dipak Manna, Upinder Singh

**Affiliations:** 1 Division of Infectious Diseases, Department of Internal Medicine, Stanford University School of Medicine, Stanford, California, USA; 2 Department of Microbiology and Immunology, Stanford University School of Medicine, Stanford, California, USA

**Keywords:** Entamoeba, amoebiasis, parasite, protozoa

## Abstract

In recent years, substantial progress has been made in understanding the molecular and cell biology of the human parasite *Entamoeba histolytica*, an important pathogen with significant global impact. This review outlines some recent advances in the *Entamoeba* field in the last five years, focusing on areas that have not recently been discussed in detail: (i) molecular mechanisms regulating parasite gene expression, (ii) new efforts at drug discovery using high-throughput drug screens, and (iii) the effect of gut microbiota on amoebiasis.

## Introduction

*Entamoeba histolytica* is a unicellular protozoan parasite transmitted to humans via contaminated food or water and is the causative agent of amoebiasis ^1, 2^. The infectious cycle begins with ingestion of the cyst, which is able to survive in the environment outside the human host ^3, 4^. After ingestion, parasites excyst as trophozoites in the small intestine and eventually reach the colon, where they adhere to colonic mucins and epithelial cells. The most common disease attributed to *E. histolytica* is amoebic colitis, whose symptoms range from asymptomatic colonization of the colon to severe, bloody diarrhea. In a subset of patients, extra-intestinal disease occurs following dissemination of the parasite to the liver, lung, or brain ^5^. Owing to unknown factors, some trophozoites encyst, allowing them to be excreted in the stool and to go on to infect new hosts. Although *E. histolytica* encysts naturally inside the human host, no culturing method has been able to induce encystation in laboratory conditions. However, *Entamoeba invadens*, a reptilian amoeba, can successfully be induced to undergo encystation in the laboratory and is the accepted model to study amoebic stage conversion *in vitro*.

Amoebiasis is widespread among infants in developing countries because of poor hygiene and sanitation, contamination of food and water with feces, and malnourishment ^6– 8^. For instance, in an urban slum of Dhaka, Bangladesh, one-third of infants are infected with *E. histolytica* within the first year of life ^8– 10^. Consequently, amoebiasis prevalence is higher in developing countries, such as the Indian subcontinent, tropical and central regions of Africa, and South America ^11, 12^. However, recent reports also identified amoebic infections in east Asian developed countries and Australia ^13– 16^. In developed countries, *E. histolytica* infection is typically seen in new immigrants and travelers returning from regions where amoebiasis is endemic, and in Japan there is a relatively high incidence of disease in homosexual men ^13, 15– 17^. These findings suggest that amoebiasis can also be a re-emerging disease in developed countries.

In this review, we will highlight a few topics that have emerged in the study of *Entamoeba* in the last five years. A number of excellent reviews have recently been published on the immune response to *Entamoeba* ^18^, immune evasion mechanisms by the parasite ^19^, trogocytosis (nibbling of host cells by the amoeba) ^20– 22^, and virulence determinants ^23^. Thus, we will focus on topics for which recent reviews are not available.

## Molecular methods regulating parasite gene expression

### Elucidating the molecular mechanisms regulating gene expression

Regulation of gene expression is a complex process requiring the simultaneous coordination of large-scale cellular processes (for example, DNA replication and chromosomal segregation) as well as more local processes (for example, euchromatin stabilization and RNA polymerase recruitment). The processes governing transcription and gene expression in *Entamoeba* remain poorly understood. Recent efforts have elucidated mechanisms for stabilizing ribosomal RNA during encystation ^24^ as well as under stress conditions ^25^. Forward-genetic screens have helped determine the target genes regulated by specific signaling transduction pathways ^26^. Additionally, transcriptome analyses in *Entamoeba* have helped to identify cis-elements and trans-acting factors involved in regulating gene expression ^27, 28^. However, despite ongoing efforts, only a handful of DNA motifs and transcription factors have thus far been characterized ^29^.

In a follow-up to their initial report characterizing the transcription factor EhPC4 ( *E. histolytica* positive cofactor 4) and its role in regulating the expression of genes involved in cell migration ^30^, Hernández de la Cruz *et al*. recently identified a new role for EhPC4 in regulating DNA replication and genome stability ^31^. *E. histolytica* trophozoites exist in cultures as polyploid cells (a subpopulation of cells having either a single polyploid nucleus or multiple nuclei), whereas cysts contain four haploid nuclei. In trophozoites, heterogeneous DNA content is due to genome re-duplication and uncoupling of nuclear division and cytokinesis ^32, 33^. Therefore, the recent data presented by Hernández de la Cruz *et al*. are important because they are among the first to identify a protein involved in polyploidy and genetic heterogeneity in *Entamoeba*. The complexity of DNA organization and structure in *Entamoeba* has posed a challenge toward unraveling aspects of parasite biology that regulate the flow of information, which arguably influences all other aspects of parasite biology (that is, metabolism and development). Importantly, polyploidy has posed some limitations on parasite genetic engineering, and further molecular dissection of this pathway could aid in the development of improved genetic tools, which can be applied to the study of parasite biology.

### Advances in amoebic RNA interference and gene regulation

The RNA interference (RNAi) pathway is an important basic biological process for regulating gene expression and genome stability as well as a robust tool for genetic manipulation ^34– 36^. Multiple pathways exist for biogenesis and function of small RNAs; however, all mature small RNAs ultimately associate with an Argonaute (Ago) protein to form an RNA-induced silencing complex, which mediates gene silencing ^37– 39^. Silencing occurs via target RNA cleavage, translational repression, or transcriptional gene silencing (TGS) ^40^. In the case of TGS, RNAi components mediate gene silencing by recruiting histone modification enzymes to targeted loci. Post-translational modifications of the amino terminal tails of histones alter the condensation state of chromatin, regulating the accessibility of DNA-binding sites for components of the transcriptional machinery ^41^.

Studies in model systems have provided much of what is known about RNAi ^42, 43^, although data from non-model organisms have uncovered important variations ^44– 46^. *E. histolytica* has a robust and non-canonical endogenous RNAi pathway, which regulates gene expression ^44, 47^. *Entamoeba* has an abundant population of 27nt small RNAs that have 5′-polyphosphate (polyP) termini, indicating that they are not Dicer products—an observation made only in amoeba, *Caenorhabditis elegans*, and parasitic nematodes ^43, 44, 48^. The repertoire of non-canonical RNAi proteins was recently expanded with the characterization of EhRNaseIII, a minimal and non-canonical Dicer-like protein in *E. histolytica*. Having a single RNaseIII domain and devoid of all domains typically associated with Dicer enzymes in other systems, EhRNaseIII is capable of processing double-stranded RNA into smaller RNA fragments that productively contribute to gene silencing ^49^.

Investigating the endogenous RNAi pathway in *Entamoeba*, Morf *et al*. ^50^ discovered that a gene to which abundant small RNAs map can “trigger” silencing of other genes fused to it ^51^. This has been an important advance for the community, as methods for genetic manipulation in *Entamoeba* have previously been limited and technically challenging. Recently, the trigger-silencing approach was adapted for use in *E. invadens*, which should allow this to be a robust system for genetic analyses of developmental pathways and developmental control ^52^. More importantly, in *E. histolytica*, the trigger-silencing approach was used to demonstrate that RNAi-mediated gene silencing in amoeba involves repressive epigenetic histone modifications ^53^. Future efforts to dissect the machinery responsible for the RNAi and trigger-gene silencing will be important as a means to improve and refine the silencing methodology and to identify aspects of the machinery that may be novel to *Entamoeba*.

### Mechanistic understanding of transcriptional gene silencing in *Entamoeba*

It has been demonstrated in *E. histolytica* that genes targeted by small RNAs are effectively silenced. The ability to silence genes in *E. histolytica* was accidentally discovered following efforts to overexpress an amoebapore gene ( *Ehap-a*); this strain, in which *Ehap-a* is permanently silenced, is the G3 clone ^54^. However, the mechanism by which TGS is initiated and maintained in *Entamoeba* remained unclear. Huguenin *et al*. first proposed a role for chromatin remodeling in the regulation of gene expression in the G3 clone of *Entamoeba* ^55^. They reported a decrease in methylation of lysine 4 of histone 3 (H3K4) and an overall enrichment of H3 near transcriptionally silent loci. Subsequent analyses revealed that the 27nt small RNAs mediated the silencing of the *Ehap-a* gene observed in the *Entamoeba* G3 clone ^48^. The small RNAs were shown to have nuclear localization, and an enrichment of EhAgo2-2 was found in close contact with silenced gene loci ^48^.

Recently, Foda *et al*. identified the first epigenetic histone modification involved in RNAi-mediated TGS in *E. histolytica* ^53^. Using a trigger expression vector to induce the expression of small RNAs to a transcriptionally active gene, they observed dimethylated H3K27 (H3K27Me2) deposition at both episomal and chromosomal gene copies. Importantly, their model links RNAi gene silencing and repressive histone-mediated TGS in *Entamoeba*. Additionally, other active and repressive epigenetic modifications for *Entamoeba* histones have been described. Interestingly, unlike most eukaryotes, both activating and repressive post-transcriptional histone modifications co-localized, suggesting that the nuclear organization of *Entamoeba* is atypical ^56^. This is not surprising and is consistent with observations that *Entamoeba*’s replication cycle and chromatin organization are characteristically unique ^57^.

### Toward identifying the biological significance of RNA interference in *Entamoeba*

One aspect that continues to elude clarification is the biological impact of the RNAi pathway in *Entamoeba*. In other systems, RNAi is reported to regulate diverse biological processes ^58^. In *Entamoeba*, the RNAi pathway has been reported to silence genes relevant to virulence and thus contributes to strain-specific virulence profiles ^59^. In an attempt to identify the biological conditions under which gene expression is regulated by RNAi, Zhang *et al*. ^60^ investigated the possibility that RNAi in *Entamoeba* is involved in regulating gene expression in response to oxidative or heat stress or in parasite stage conversion.

In their report, Zhang *et al*. analyzed small RNA profiles from *E. histolytica* trophozoites subjected to oxidative stress or heat shock. Although robust small RNA populations are present in each condition, the small RNA populations and the genes to which they map did not change abundance or expression under the various stress conditions. To determine whether the small RNAs controlled gene expression relevant to stage conversion, they generated and sequenced 27nt small RNA libraries from encysting and excysting *E. invadens* parasites. Similar to *E. histolytica*, genes targeted by small RNAs in *E. invadens* are silenced. However, somewhat unexpectedly, they demonstrated that the 27nt small RNAs do not appear to regulate genes that change expression during stage conversion ^60^. Adding to the genomic complexity already observed in *Entamoeba*, there are some notable differences in the small RNA profile between *E. histolytica* and *E. invadens*. *E. invadens* had a larger percentage of small RNA reads mapping to intergenic regions, retrotransposons, and repetitive elements while having a smaller percentage of small RNA reads mapping to open reading frame. Zhang *et al*. speculated that the large percentage of small RNAs that map to retrotransposons and repetitive elements suggests that RNAi in *E. invadens* may have roles in preserving genome integrity and stability.

Thus, despite extensive efforts, to date no biological condition(s) that are regulated by RNAi in *Entamoeba* have been identified. However, maintenance of the pathway across multiple *Entamoeba* species and the conservation of genes silenced by RNAi indicate a strong selection pressure and an important biological role for this phenomenon in amoebae. It is interesting to speculate that, in this unique parasite, RNAi is central to the flow and regulation of information that go beyond those involved in transcriptional silencing. Growing evidence in fungi, plants, and animals suggests that RNAi plays important roles in regulating numerous nuclear processes, including transposon regulation, heterochromatin formation and propagation, and genome stability ^61^. Additionally, it is possible that amoebic small RNAs serve to mediate intercellular communication via their transfer in exosomes or extracellular vesicles or both, as noted in the parasitic nematode *Heligmosomoides polygyrus* ^62^. Given that the parasite maintains a complex and robust endogenous RNAi pathway, there is no doubt that much is yet to be uncovered about its impact on parasite biology.

The regulation of biological processes by RNAi does not need to be confined exclusively to small RNAs. Emphasis thus far has been on the 27nt small RNA population in *Entamoeba*. However, diverse non-coding RNA species have been found to regulate biological processes in other systems, such as the discovery that long non-coding RNAs (lncRNAs) function as scaffolds to regulate the expression of a large subset of genes in mammals ^63^ and are centrally involved with X chromosome inactivation ^64, 65^. As such, there exists a need to expand the breadth and scope of investigations for amoebic processes that are potentially regulated by other non-coding RNA species. As an example, cell division in *Entamoeba* is poorly understood, especially related to our understanding of the mechanism regulating chromatin pairing and segregation during cell division. *Entamoeba* exist as multinucleated polyploid cells *in vitro* and *in vivo* ^66^, suggesting that the parasites are able to mitigate issues arising due to gene copy number. Given the atypical cell cycle in *Entamoeba* ^33^, one could be enticed to hypothesize that amoebic RNA is involved in genome stabilization during cellular division. Consistent with this hypothesis, growing evidence suggests that long and small RNAs can serve as an alternative to DNA-binding proteins for epigenetic regulation of gene expression ^58^. Notably, the Bhattacharya group discovered the first lncRNA in *Entamoeba* ^67^ and described its role in mediating stress responses. Further research on amoebic lncRNA is poised to help address remaining questions in the field.

## New drugs against amoebiasis

At present, there are no vaccines available against amoebiasis. Currently, metronidazole is the drug of choice and is used worldwide to treat invasive amoebiasis in both adults and children ^68– 71^. In addition, the luminal amoebicide paramomycin is administered to eradicate cysts from the colon ^69, 70^. Other nitroimidazole derivatives such as tinidazole and ornidazole have improved dosing schedule with a single 2 g dose once daily for three days ^69, 70^. Marie and Petri highlighted the use of available anti-amoebic drugs by comparing their efficacy and safety ^11^. However, metronidazole has significant associated side effects, including nausea, vomiting, and headaches; furthermore, it has been found to be mutagenic in bacteria and carcinogenic in experimental mammalian models at high doses over long periods ^72, 73^. Thus, there is an important need for the development of specific, novel, and safe drug(s) to treat amoebiasis.

In an important advance in this direction, Debnath *et al*. developed an automated high-throughput drug screen and discovered that auranofin is potent against *E. histolytica* trophozoites both *in vitro* and *in vivo* and also is effective against *E. invadens* cysts ^74– 76^. Auranofin is a US Food and Drug Administration-approved oral, gold-containing drug that has been in clinical use to treat rheumatoid arthritis for the last 25 years ^76^. It is reported to be effective against several other protozoan parasites ^77^ such as *Giardia lamblia* ^78^, *Trypanosoma brucei* ^79^, and *Plasmodium falciparum* ^80^ and kills the promastigote stage of *Leishmania infantum* ^81^ *in vitro*. It also kills other human parasites, such as *Schistosoma mansoni*, which causes the disease schistosomiasis ^77^, and also is effective against larval worms *Echinococcus granulosus* ^82^. Oral administration of auranofin significantly decreased the parasite number in both the amoebic colitis and the liver abscess models, suggesting its great potential as an anti-amoebic drug ^74, 83^. Transcriptional profiling identified *E. histolytica* thioredoxin reductase (EhTrxR) protein as a target of auranofin. It is proposed that a monovalent gold atom, Au, is released from auranofin, subsequently inactivating the EhTrxR protein, which interferes with the redox homeostasis in the parasite and subsequently makes the parasites more sensitive to reactive oxygen-mediated cell killing ^84^. Jeelani and Nozaki recently highlighted this unique thiol-based redox metabolism system as a drug target against amoebiasis ^85^. *E. histolytica* trophozoites are microaerophilic in nature and prefer the microaerobic environment in the lumen of the large intestine. However, once trophozoites invade the tissue barrier, they are exposed to a high-oxygen environment ^86^. Being a microaerophilic organism, *E. histolytica* does not contain most of the elements required for eukaryotic oxidative stress defense systems, including catalase, peroxidase, glutathione, and glutathione-recycling enzymes ^85, 87, 88^. However, *Entamoeba* has several other key redox regulators, which are unique to *Entamoeba* and which show promise as drug targets ^85^. In another approach, Boyom *et al*. repurposed the open access malaria box compounds and identified effective compounds against *E. histolytica* ^89^, although further characterization and validation are needed before they can be identified as valid drug candidates against amoebiasis.

Efforts have been made by several groups to develop anti-amoebic drugs from natural resources, particularly from plants or herbs which were used in traditional remedies against amoebic infection ^90– 92^. Recent screening of a natural products library from fungi and actinomycetes sources against *E. histolytica* identified several cysteine synthase inhibitors ^93^. However, the toxicity of these compounds against human cell lines precluded their use as an alternate to metronidazole.

In another approach, Shahinas *et al*. used a target-based drug screen for *Entamoeba* heat shock protein 90 (HSP90) inhibitors and identified five compounds (rifabutin, rutilantin, cetylpyridinium chloride, pararosaniline pamoate, and gentian violet), which inhibited *E. histolytica* growth in the micromolar range ^94^. HSP90 is an essential chaperone-like protein and a good drug target candidate as the N-terminal ATP-binding domain is structurally diverse and presumably specific for individual organisms. Thus, inhibitors that are highly functional against *Entamoeba* HSP90 could be highly unique and specific. 

The recent interest and efforts to identify new compounds against *Entamoeba* are important advances. Ideally, drugs that target multiple microaerophilic protists could be identified (as with the recent identification of auranofin) ^74^, as a one-drug/multiple-bug approach is most likely to be effective, especially in developing countries where disease burden is high and resources are limited.

## Effect of gut microbiota in amoebiasis

With the recent explosion of data on the human microbiome, it makes sense to dissect the interplay between *Entamoeba*, a gut resident, with the human colonic microbiome. Epidemiological data suggest that over 500 million people are infected by *E. histolytica* worldwide ^95^. Interestingly, not all individuals are equally susceptible to *Entamoeba* infection; 90% of the infected individuals are asymptomatic carriers, whereas the remaining 10% show serious intestinal and extra-intestinal diseases such as colitis, dysentery, and amoebic liver abscesses ^1^. One possible explanation for this observation is the difference in immunity and gut microbiota between individuals. Recently, Burgess and Petri extensively reviewed the role of microbiota in *E. histolytica* infection ^96^. It was shown that the severity of amoebic colitis was influenced by different factors such as nutrition and maternal breast milk IgA antibody level against *Entamoeba* lectin ^97^. Earlier studies by Phillips *et al*. revealed the importance of microbiota and their role in *E. histolytica* infection ^98, 99^. They found that none of the germ-free animals inoculated with *E. histolytica* developed amoebic lesions; however, most of the conventional animals that presumably have gut microbiota developed amoebic ulcers ^98^. Independent studies by Rani *et al*. ^100^ and Reyna-Fabian *et al.* ^101^ demonstrated a significant role of gut microbiota and its influence in patients with amoebic liver abscesses.

More recently, Burgess *et al*. demonstrated that introduction of commensal bacteria alters the mucosal immune system and reduces the susceptibility of mice to amoebic infection ^102^. Thus, alteration of the gut microbiota provides protection against infection by *E. histolytica* ^102^. In a separate report, an association of anaerobic Gram-negative bacteria, *Prevotella copri*, and *Entamoeba* was observed in infected children ^97^. *P. copri* is associated with gut inflammation and the generation of excessive immunity in patients and in animal models, which may facilitate the ability of *Entamoeba* to establish a productive infection and influence the progression to invasive disease ^97, 103^. These data suggest that *E. histolytica* infection is influenced by the inflammatory state of the gut, which is potentially associated with changes in the gut microbiome. These studies may provide insights into why some patients get invasive disease, the factors that contribute to parasite-associated malnutrition and growth inhibition, and how intestinal parasites may change gut flora and impact the systemic immune response.

## Summary

In this review, we have highlighted recent advances that have contributed to improving our understanding of biological processes central to the human pathogen *E. histolytica*. A better understanding of the molecular mechanisms regulating gene expression have been achieved, aided in part by the development of the RNAi trigger-silencing approach. The emerging role of gut microbiota in amoebiasis has only begun to be addressed, leaving many tantalizing questions to be answered. In addition, the repurposing of compounds to identify new drugs against *E. histolytica* shows great potential to improve the treatment of amoebiasis, a neglected tropical disease. Given the many unique aspects of this parasite’s biology and its ongoing impact on human health, much remains to be resolved.
